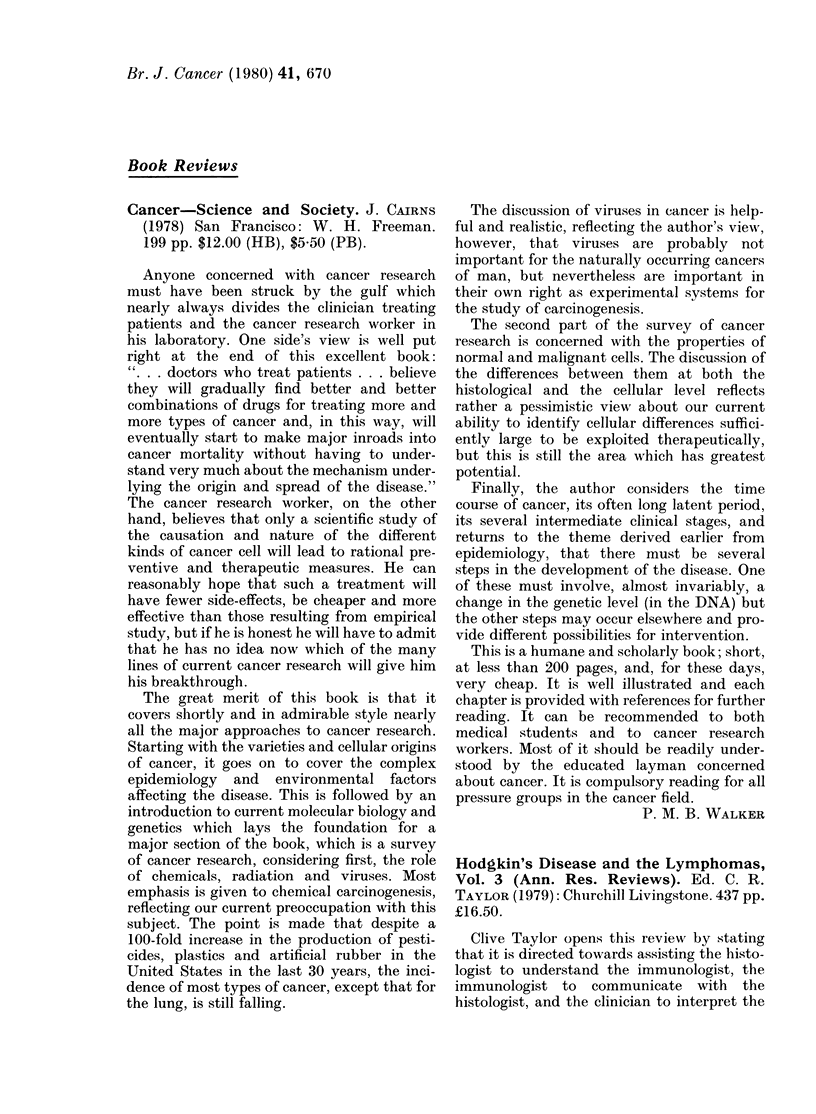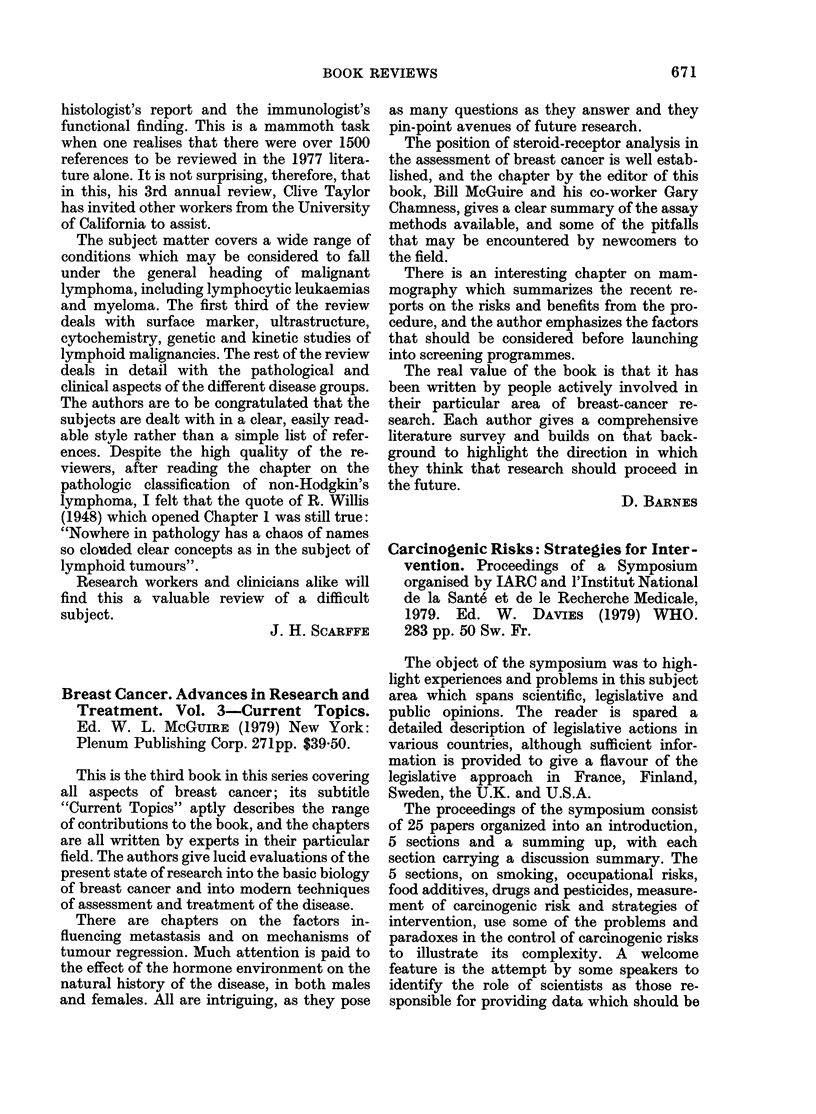# Hodgkin's Disease and the Lymphomas, Vol. 3 (Ann. Res. Reviews)

**Published:** 1980-04

**Authors:** J. H. Scarffe


**Hodgkin's Disease and the Lymphomas, Vol. 3 (Ann. Res. Reviews).** Ed. C. R. Taylor (1979): Churchill Livingstone. 437 pp. £16.50.

Clive Taylor opens this review by stating that it is directed towards assisting the histologist to understand the immunologist, the immunologist to communicate with the histologist, and the clinician to interpret the histologist's report and the immunologist's functional finding. This is a mammoth task when one realises that there were over 1500 references to be reviewed in the 1977 literature alone. It is not surprising, therefore, that in this, his 3rd annual review, Clive Taylor has invited other workers from the University of California to assist.

The subject matter covers a wide range of conditions which may be considered to fall under the general heading of malignant lymphoma, including lymphocytic leukaemias and myeloma. The first third of the review deals with surface marker, ultrastructure, cytochemistry, genetic and kinetic studies of lymphoid malignancies. The rest of the review deals in detail with the pathological and clinical aspects of the different disease groups. The authors are to be congratulated that the subjects are dealt with in a clear, easily readable style rather than a simple list of references. Despite the high quality of the reviewers, after reading the chapter on the pathologic classification of non-Hodgkin's lymphoma, I felt that the quote of R. Willis (1948) which opened Chapter 1 was still true: "Nowhere in pathology has a chaos of names so clouded clear concepts as in the subject of lymphoid tumours".

Research workers and clinicians alike will find this a valuable review of a difficult subject.

J. H. Scarffe